# Aphids and Ants, Mutualistic Species, Share a *Mariner* Element with an Unusual Location on Aphid Chromosomes

**DOI:** 10.3390/genes12121966

**Published:** 2021-12-09

**Authors:** Jesús Vela, Eugenia E. Montiel, Pablo Mora, Pedro Lorite, Teresa Palomeque

**Affiliations:** Department of Experimental Biology, Genetics Area, University of Jaén, Paraje las Lagunillas s/n, 23071 Jaén, Spain; jvela@ujaen.es (J.V.); emontiel@ujaen.es (E.E.M.); pmora@ujaen.es (P.M.); tpalome@ujaen.es (T.P.)

**Keywords:** ants, aphids, *mariner* elements, rDNA genes, NOR, horizontal transfer events

## Abstract

Aphids (Hemiptera, Aphididae) are small phytophagous insects. The aim of this study was to determine if the *mariner* elements found in the ant genomes are also present in *Aphis fabae* and *Aphis hederae* genomes and the possible existence of horizontal transfer events. Aphids maintain a relationship of mutualism with the ants. The close contact between these insects could favour horizontal transfer events of transposable elements. *Myrmar mariner* element isolated from *Myrmica ruginodis* and *Tapinoma ibericum* ants have also been found in the two *Aphis* species: *A. fabae* and *A. hederae* (*Afabmar-Mr* and *Ahedmar-Mr* elements). Besides, *Afabmar-Mr* could be an active transposon. *Myrmar*-like elements are also present in other insect species as well as in one Crustacean species. The phylogenetic study carried out with all *Myrmar*-like elements suggests the existence of horizontal transfer. Most aphids have 2n = 8 with a XX-X0 sex determination system. Their complicated life cycle is mostly parthenogenetic with sexual individuals only in autumn. The production of X0 males, originated by XX females which produce only spermatozoa with one X chromosome, must necessarily occur through specialized cytogenetic and molecular mechanisms which are not entirely known. In both aphid species, the *mariner* elements are located on all chromosomes, including the X chromosomes. However, on the two X chromosomes, no positive signals are detected in their small DAPI-negative telomere regions. The rDNA sites are located, as in the majority of Aphids species, on one of the telomere regions of each X chromosome. The hybridization patterns obtained by double FISH demonstrate that *Afabmar-Mr* and *Ahedmar-Mr* elements do not hybridize at the rDNA sites of their host species. Possible causes for the absence of these transposons in the rDNA genes are discussed, probably related with the X chromosome biology.

## 1. Introduction

Aphids (Aphididae) are small phytophagous insects. Many species represent important crop pests that may cause damage and lower agricultural yields. Aphids frequently establish mutualistic relationships with ants, a behaviour that has been widely studied [1,2,3]. Mutualism is advantageous for both partners; aphids provide ants with sugar-rich honeydew as a source of food and the ants protect the aphids against various natural enemies and improve the hygiene of the aphid colony. On the other hand, aphids can damage crops in other ways; in fact, aphids are considered the major vectors of plant viruses [4,5]. Different aphids´ virus and fungus have been found in ants [6].

Cytogenetically, aphids are characterized by the presence of holocentric (or holokinetic) chromosomes without a defined centromere. In this type of chromosomes, the spindle fibres adhere along the entire length of the chromosomes. Holocentric chromosomes have also been found in some groups of plants, insects, arachnids, and nematodes. Generally, species with holocentric chromosomes show an inverted meiotic sequence, which implies that homologs segregation does not happen until the second meiotic division [7]. Aphids have a life cycle characterized by the rapid succession of parthenogenetic generations formed exclusively by XX females. The bisexual reproduction, with X0 males and XX females, constitutes only a short part of their life cycle, coinciding with the shortening of the photoperiod in autumn. Mating between them produces overwintering resistant eggs that will later originate parthenogenetic females. Parthenogenetic XX females generate males and females. This process must necessarily occur through specialized cytogenetic mechanisms, which are poorly understood. During the embryonic development of female aphids, the division of oogonias originates a germarium, formed by 32 undifferentiated oogonial cells. A cell begins its development as an oocyte and enters in the growth phase. The process continues with the maturation division with the formation of a polar body, and the reestablishment of the diploid chromosomal number. During the process, the chromosomal behaviour will be different depending on whether it follows a parthenogenetic development or a sexual cycle. If the bisexual cycle begins, the two X chromosomes appear end-to-end paired by the time the oocyte growth phase begins. This union between X chromosomes appears to be essential for sex determination. If the oocytes follow a female developmental pattern, the pairing between X chromosomes will be lost before the maturation division. If the oocytes follow a male development the two X chromosomes remain paired until the end of the process, so that only one X chromosome is preserved, whereas the other is eliminated in the polar body [8,9,10,11,12,13]. It is debated whether the missing X chromosome is random or not [8,14,15]. The molecular mechanisms that induce the formation of sexual phenotypes, are still unknown [16], although multiple genes that are differentially expressed in males, in sexual females and in parthenogenetic females, have been studied [17,18,19]. It has been suggested that aphids have a high rate of evolution through chromosomal rearrangements processes [12,17,19]. The stabilization and inheritance of chromosomal rearrangements could be facilitated in organisms with holocentric chromosomes, since the acentric fragments, would probably be lost in organisms with a localized centromere, but they could be adequately inherited in aphids [9,12]. Recently, Mathers et al. [19], by chromosome-scale genomic assembly technology, confirmed the high rate of chromosomal rearrangements in the evolution of autosomes. On the contrary, and according to the same authors and others [17,20], the X chromosome shows long-term high structural conservation.

Transposable elements (TEs) are repeated DNA segments with the ability to move from one locus to another within the host genome. Eukaryotic TEs have been classified into two categories, class I (or retrotransposons) and class II (or DNA transposons) based especially on their structure and mechanism of transposition [21]. *Mariner* elements are class II TEs. The first *mariner* element described was *Mos1*, isolated from *Drosophila mauritiana* [22]. *Mariner*-like elements show similar motifs and belong to the *Tc1-mariner* family, a member of the *IS630-Tc1-mariner* superfamily, present in most of the eukaryotic and prokaryotic species [23,24]. 

Horizontal transfer (HT) events allow the invasion of new genomes by a transposable element. Horizontal transfer consists of the passage of DNA from one organism to another through a mechanism other than reproduction. In recent years, it has been shown that HT is a much more common event than previously considered, with great importance in the evolution of eukaryotic genomes [25,26,27,28]. Likewise, numerous HT events have been described between genomes of different ant species and between ants and other insect species [29,30,31]. A key question about HT among eukaryotes is the knowledge of the vectors that could intervene in this transfer. Species involved in parasitism, mutualism or similar interrelationships could be good candidates to be possible vectors. In this sense, Filée et al. [32] have suggested that *Rhodnius prolixus*, a vector of *Trypanosoma cruzi* that causes Chagas disease, could be responsible for the horizontal transfer of transposable elements between species on which they are fed. Different authors have proposed that viruses could be the vectors for horizontal transfer events, since they inject their genomes into host cells, replicate in them and can be transmitted horizontally between their hosts [33,34].

Four full-length *mariner* transposable elements have been described in ant genomes, all of them belonging to the *Tc1-mariner* superfamily. Three of these *mariner* elements belong to the *mauritiana* subfamily. Initially, each one was isolated from a different ant genus of the subfamily Myrmicinae; *Sinvmar* was isolated from several *Solenopsis* species [35], *Myrmar* from *M. ruginodis* [36,37] and *Mboumar* from *Messor bouvieri* [38]. *Azteca*, the fourth *mariner* element, belonging to the *irritans mariner* subfamily, was initially found in *T. ibericum* (subfamily Dolichoderinae) [29,30]. Subsequently, it has been shown that these *mariners* are widespread in several genera from different ant subfamilies [30,31]. The objective of this study is to determine if the *mariner* elements found in ants are present in aphids, specifically in *A. fabae* and *A. hederae*. Aphids maintain a relationship of mutualism with ants. The close physical contact between these insects could favour HT events of transposable elements, without excluding the possible intervention of virus or other vectors in these events. 

## 2. Materials and Methods

### 2.1. Material, DNA Extraction, PCR Amplification and Cloning

Samples of *A. hederae* were collected on ivy (*Hedera helix*) plants on the campus of the University of Jaén (Spain). The *A. fabae* specimens were collected in a broad bean (*Vicia faba*) crop in Jaén. Adult females were kept alive in the laboratory for the preparation of chromosome spreads. Other females were preserved in 100% ethanol at −20 °C and used subsequently for genomic DNA extraction. Total genomic DNA was isolated using the NucleoSpin Tissue kit (Machery-Nagel GmbH & Co., Düren, Germany) following the instructions provided by the company. 

PCR was used to test the presence of the ant transposons in the aphid genomes, using specific primers designed for each transposon based in the inverted terminal repeats sequences (ITRs) (Supplementary Table S1): *Myrmar* [29,36,37], *Mboumar* [38], *Sinvmar* [29,35] and *Azteca* [30]. PCR amplifications were initially denatured at 92 °C for 2 min and then, subjected to 30 cycles at 92 °C (30 s), 50 °C (30 s), 72 °C (2 min), with a final elongation step of 72 °C for 5 min. Reactions were set up in a 50 μL mixture containing 100 ng of genomic DNA, 0.5 mM dNTPs, 50 pmol of primers and 1 U of Taq polymerase (Bioline, London, UK). The amplified fragments were analysed by electrophoresis in 1% agarose gels, eluted from agarose gel and cloned into the pGEM-T Easy vector (Promega, Southampton, UK). Recombinant plasmids were sequenced on both strands by the dideoxy sequencing method.

### 2.2. Sequence Analyses and Molecular Evolutionary Analyses

Multiple-sequence alignments of the *Myrmar*-like element were performed using Clustal Omega from EMBL-EBI [39] (https://www.ebi.ac.uk/Tools/msa/clustalo/ accessed on 11 November 2021). Sequence comparisons, open reading frame (ORF) searches, and other sequence analyses were performed using available online programs from NCBI (http://www.ncbi.nlm.nih.gov/guide/, accessed on 11 November 2021). The evolutionary divergence between sequences was evaluated using MEGA version X [40]. The nucleotide substitution models were evaluated using MEGA. The model with the lowest Bayesian Information Criterion (BIC) score was considered the best for describing the substitution pattern. The NSP@Network Protein Sequence Analysis program was used for the prediction of he-lix-turn-helix motifs [41]. 

The searches for sequences with homology to those isolated from *A. fabae* and *A. hederae* were carried out using Repbase (http://www.girinst.org/, accessed on 22 October 2021), the GenBank/NCBI DNA databases using the BLAST network service (https://blast.ncbi.nlm.nih.gov/Blast.cgi, accessed on 22 October 2021), and “The aphid genome database” (AphidBase https://bipaa.genouest.org/is/aphidbase/, accessed on 22 October 2021). The default options were used in all BLAST searches. Only hits with a BLAST output of e-value = 0.0 were considered. These hits correspond to the highest score values found.

The phylogenetic relationships among the *Myrmar*-like elements were analysed using maximum-likelihood (ML) methods with the T92+G model using the MEGA X program. Bootstrap values for each branch were assessed from 1000 replicates. We used the sequences *TnigmarMb-1* and *Mboumar-9 mariner* elements isolated from T. *ibericum* and *M. bouvieri* ants as out-groups, both of which belong to the *mauritiana* subfamily. Material previously analysed as *Tapinoma nigerrimum* is currently considered as *T. ibericum* [42]. Consequently, the name *T. ibericum* was used in this paper. However, we will continue to call the *mariner* sequences of this species as *Tnigmar-Mb* and *Tnigmar-Mr*, according to their names in the GenBank database. We also used *Mos1* as an out-group, which is the best-known element of the *mauritiana* subfamily.

### 2.3. Chromosome Preparation, Ag-Stain Technique and In Situ Hybridization Procedures

Chromosome slides were performed according to the method described by Manicardi et al. [43], using adult females. The chromosomes were stained with DAPI (4′-6-diamino-2-fenil-indol), or Giemsa, and analysed with an Olympus (Hamburg, Germany) BX51 fluorescence microscope equipped with an Olympus DP70 camera. Images were processed using Adobe Photoshop CS4 (Adobe Systems, San Jose, CA, USA). Silver nitrate staining to evidence the nucleolar organizer regions (NORs), chromosome regions containing the rDNA genes, was performed according to the technique described by Rufas et al. [44]. 

Chromosomal location of the Aphid *mariners* and the rDNA clusters was determined by fluorescent in situ hybridization (FISH). *AfabmarMr-50* and *AhedmarMr-700* were used as probes for the physical location of *mariner* transposable elements. For rDNA location by FISH, the plasmid pDmra.51#1, with a noninterrupted 11.5 kb rDNA unit of *Drosophila melanogaster* [45], was used as a probe. The probes were labelled with biotin-16-dUTP or with digoxigenin-11-dUTP using the nick translation kit (Roche Diagnostics GmbH, Mannheim, Germany). FISH was carried out following the procedure described by Palomeque et al. [46] using the digoxigenin-labelled probe (5 ng/mL in 50% formamide) and the biotin-labelled probe (2 ng probe/mL, 50% formamide). The fluorescent immunological detection was carried out using the avidin-FITC/anti-avidin-biotin system (Vector Laboratories, Burlingame, CA, USA) or an anti-DIG-rhodamine antibody (Roche Diagnostics GmbH). Slides were mounted in Vectashield with DAPI (Vector Laboratories).

## 3. Results

### 3.1. Isolation of Mariner Elements from A. fabae and A. hederae Genomes, Sequence and Phylogenetic Analyses

Several series of PCR amplification tests were carried out in order to amplify in the genome of *A. fabae* and *A. hederae* the four types of *mariner* elements found in ants. However, sequence amplification has been successful only when specific primers for the *Myrmar mariner* have been used. In both species, PCR amplification with this primer generated two bands with sizes similar to those expected for *mariner* elements (Supplementary Figure S1). Both bands were eluted from the agarose, cloned, and sequenced. Sequence analysis allowed determining that both bands showed similarity with the *Myrmar mariner* elements. Applying the nomenclature proposed by Robertson and Asplund [47] and Lorite et al. [29] the *mariner* elements were named as *Afabmar-Mr* and *Ahedmar-Mr*, respectively (Genbank accession no. OL441193-OL441226).

The sequence analysis from the cloned PCR products isolated from *A. fabae* showed that the amplified bands were 1282 bp and 1223 bp in length, respectively. The 1282-bp sequences represent full-length copies of the *mariner*, whereas the 1223-bp sequences have an internal deletion at the end of the *mariner* sequence (Supplementary Figure S2). Within the first group, as an exception, *AfabmarMr-58* is 1281 bp in length because of one nucleotide deletion (526-nucleotide position). Similarly, the isolated sequences from *A. hederae* revealed two groups, with 1281 and 1223 bp in length, respectively. As in *A. fabae*, in *A. hederae* the longest sequences represent full-length copies of the *mariner*, while 1223-bp sequences have an internal deletion (Supplementary Figure S2). Internal deleted copies in both species show the same deletion (1148-1211 nucleotide positions). All 1281-bp *Ahedmar-Mr* sequences present the same nucleotide deletion as *AfabmarMr-58*. Likewise, all 1223-bp sequences from both species share 16 mutations that are not present in the 1282-bp *Afabmar-Mr* sequences nor in the 1281-bp *Ahedmar-Mr* sequence. Finally, in all groups, indels point mutations that affect one or more sequences are observed. The evolutionary divergence is 0.012 ± 0.002 for *Afabmar-Mr* and 0.011 ± 0.002 for *Ahedmar-Mr* sequences and between species is 0.013 ± 0.002. However, the evolutionary divergence for full-length and deleted sequences is clearly lower, concretely among full-length sequences 0.008 ± 0.002 and among deleted sequences is 0.005 ± 0.001. This indicates that the full-length copies of both species have a greater similarity than between full-length and internally deleted copies within the same species. The same is observed among the internally deleted copies.

All 1282-bp *Afabmar-Mr* sequences (except *AfabmarMr-58*) are potentially active copies since they show an ORF (172-1206 nucleotide position). The putative proteins conserved the D,D(34)D catalytic motif in the C-terminal domain of the transposase (Supplementary Figure S3). Equally, the two WVPHEL and YSPDL highly conserved amino-acid motifs and other features of active *mariners* as helix-turn-helix motifs (HTH) [48], are also conserved in the majority of the sequences. The conserved motifs are considered very important for the transposase activity [49]. Therefore, these sequences could encode an active transposase. The *AfabmarMr-58* sequence presents a deletion of a nucleotide (526-nucleotide position, Supplementary Figure S2) causing a premature stop codon. The same happens for the 1281-bp *Ahedmar-Mr* sequences. Finally, all 1223-bp sequences from both species are not potentially coding copies, since the deletion shared by all of them, eliminates the stop codon. 

**Figure 1:**
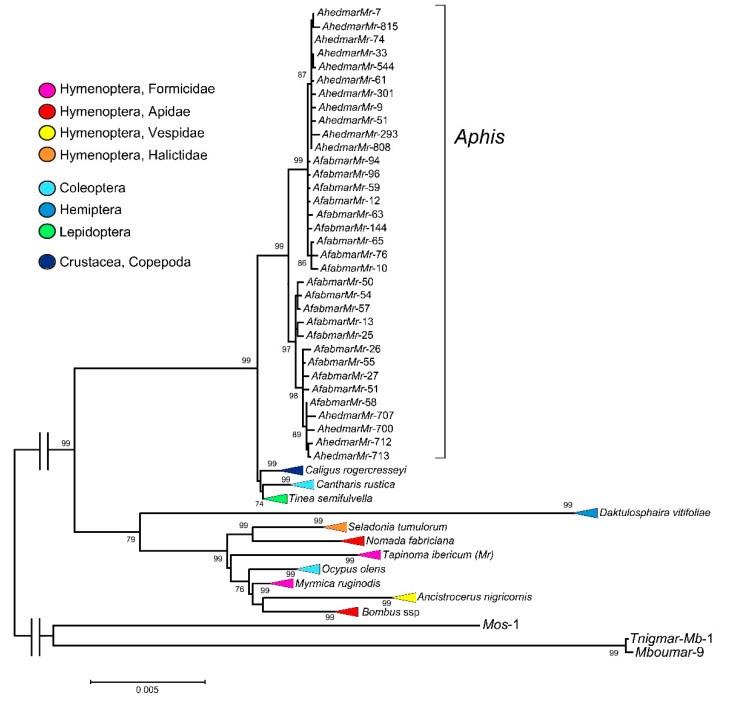
Maximum-likelihood analysis of the nucleotide *Myrmar*-like *mariner* sequences. All clades are collapsed, except those corresponding to *Afabmar-Mr* and *Ahedmar-Mr* sequences isolates from *A. fabae* and *A. hederae*. A non-collapsed phylogenetic tree is shown in Supplementary Figure S4. The access numbers and nucleotide positions of each *mariner* element are indicated in Supplementary Figure S4. Numbers indicate the bootstrap values over 1000 replications. Only bootstrap support values greater than 70% are shown.

A phylogenetic analysis was carried out using *Afabmar-Mr* and *Ahedmar-Mr* sequences (Figure 1), and all sequences included in the called “*Myrmica mariner* group” by Lorite et al. [29]. This group included *Myrmar*, *Tnigmar*-*Mr* and *Botmar* elements, isolated from *M. ruginodis* [36] and *T. ibericum* ants (Hymenoptera, Formicidae) [29], and from bumblebee *Bombus terrestris*, (Hymenoptera, Apidae) [37], respectively. In the phylogenetic tree (Figure 1 and Supplementary Figure S4), *mariner* sequences isolated from the following species have also been included: *Caligus rogercresseyi* (Crustacea, Copepoda), *Cantharis rustica* (Coleoptera, Cantharidae), *Tinea semifulvella* (Lepidoptera, Tineidae), *Daktulosphaira vitifoliae* (Hemiptera, Phylloxeridae), *Seladonia tumulorum* (Hymenoptera, Halictidae), *Nomada fabriciana* (Hymenoptera, Apidae), *Ocypus olens* (Coleoptera, Staphylinidae), *Ancistrocerus nigricornis* (Hymenoptera, Vespidae), *Bombus campestris* and *B. silvestris* (Hymenoptera, Apidae). All sequences show homology to those isolated from *A. fabae* and *A. hederae* according to the databases used. The accession number and nucleotide position of each of the sequences used in the phylogenetic tree are shown in Supplementary Figure S4. The sequences obtained using the AphidBase web server, belonging to the *D. vitifoliae* genome [50] are in Supplementary Figure S5. The phylogenetic tree shows two main clades (99 and 79 bootstrap values, respectively). All *mariner Aphis* sequences belong to the first clade and were clustered together in a highly supported clade (99 bootstrap value). These sequences are also grouped into two new different subclades. The first (99 bootstrap value) contains all internally deleted copies (1223 bp in length) from the two species, although the sequences of both species are separated since sequences of *A. hederae* are clustered together (87 bootstrap value). The second subclade (97 bootstrap value) contains all full-length copies of the *mariner*, the 1282-bp *Afabmar-Mr* and 1281-bp *Ahedmar-Mr* sequences. Again, the sequences of *A. hederae* are clustered together but a sequence from *A. fabae* (*AfabmarMr-58*) is grouped with them. Within this first main clade are also included new *Myrmar*-like sequences resulting from the database searches. These sequences belong to a beetle (*C. rustica*), a butterfly (*T. semifulvella*) and interestingly to a crustacean species (*C. rogercresseyi*). Generally, the sequences from each species are clustered together in well-supported subclades (99, 99, and 74 bootstrap values, respectively).

The *Myrmar*-like *mariners* previously described in ants and *B. terrestris* (*Botmar*) are included within the second main clade, as well new sequences resulting from the database searches, all of them belonging to insect species. Within this clade, generally all *mariner* sequences of each species are clustered together, although as an exception some *mariner* sequences from two *Bombus* species, *B. campestris* and *B. silvestris,* which are clustered together. In spite of this, the phylogeny of the *mariners* is not in concordance with the phylogeny of their hosts. We emphasize that this group includes isolated sequences from very different taxa of Hymenoptera and that their clustering does not reflect its taxonomy. For example, the isolated sequences of *Nomada* and *Bombus* genera, belonging to the Apidae family, are not clustered in the phylogenetic tree. The *mariner* sequences of the genus *Bombus* are closer to the *mariner* sequences of the genus *Ancistrocerus* belonging to the family Vespidae. Similarly, *Tnigmar* and *Myrmar*, *mariner* elements isolated from the ant genomes, neither are clustered together. Even, *Myrmar* is phylogenetically closer to *Botmar* and other isolated elements of the genus *Bombus*, than to *Tnigmar*. In addition, the subclade with sequences from a coleopteran species (*O. olens*) is intermixed with the subclades of hymenopteran species. Finally, this same clade included the *mariners* isolated from the hemipteran insect *D. vitifoliae* (Figure 1 and Supplementary Figure S4).

### 3.2. Localization of Transposable Elements by FISH

To determine the exact location of the *Afabmar-Mr* and *Ahedmar-Mr* elements, we have carried out cytogenetic molecular studies. In *A. hederae*, only the chromosome number has been studied by using the Giemsa staining technique [51,52]. In *A. fabae* the location of the NORs using silver staining techniques have also been applied [53]. Females of both species have 2n = 8, the most common chromosome number found in the genus *Aphis* [12,51,52,54]. 

**Figure 2:**
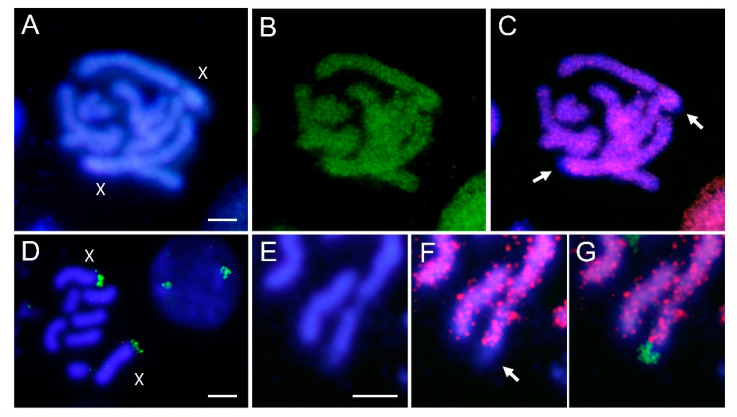
(**A**) Mitotic chromosomes of *A. fabae* after DAPI staining, (**B**) the same metaphase after FISH with biotin-labelled *Afabmar-Mr* as a probe, and (**C**) merged image. (**D**) Chromosomal localization of rDNA sites, using biotin-labelled rDNA as a probe, showing hybridization signals on one of the terminal regions of both X chromosomes (green). (**E**) Selected chromosome X after DAPI staining, (**F**) FISH with biotin-labelled *Afabmar-Mr* as a probe (red), and (**G**) digoxigenin-labelled rDNA as a probe (green). Arrows indicate the terminal DAPI negative region of the X chromosomes where is located the nucleolar organizing region. Bar =10 µm.

The analysed *A. fabae* population shows the described chromosome number for this species of 2n = 8, with six autosome pairs and two X chromosomes, differentiated by their larger size (Figure 2). Both X chromosomes stain almost uniformly with DAPI except for one of their telomeric regions (Figure 2A). Chromosomal localization of *Afabmar-Mr* elements was performed using FISH techniques. The results show that this *mariner* is present on all chromosomes, including the X chromosomes (Figure 2B). However, on the two X chromosomes, no positive hybridization signals were detected in their small DAPI negative telomeric regions (Figure 2C). FISH with rDNA as a probe showed that the NORs are located at one of the telomeric regions of both chromosomes X (Figure 2D), as was pointed using silver staining [53]. Double FISH using as probes rDNA and *Afabmar-Mr* showed that the *mariners* are not located on the NOR regions (Figure 2E–G). 

**Figure 3:**
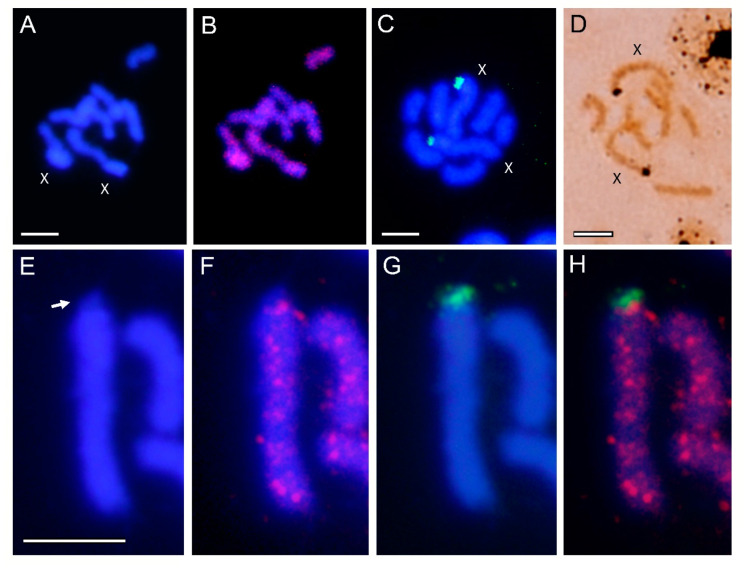
(**A**) Mitotic chromosomes of *A. hederae* after DAPI staining, (**B**) the same metaphase plate after FISH using biotin-labelled *Ahedmar-Mr* as a probe. (**C**) FISH using biotin-labelled rDNA as a probe showing hybridization signals in one of the terminal regions of both X chromosomes (green). (**D**) Silver staining of NORs, also reveals the position of the rDNA in the terminal regions of both X chromosomes. (**E**) Selected chromosome X showing DAPI staining, (**F**) the same chromosome after FISH using biotin-labelled *Ahedmar-Mr* as a probe (red), (**G**), FISH using digoxigenin-labelled rDNA as probe (green) and (**H**) merged image. The arrow indicates the terminal DAPI negative region of the X chromosomes where is located the nucleolar organizing region. Bar = 10 µm.

The chromosome number of *A. hederae* is also 2n = 8 (Figure 3A), the X chromosomes in this species also being the largest ones. FISH using *Ahedmar-Mr* as a probe showed that this *mariner* is also present on all chromosomes in this species (Figure 3B). FISH using rDNA as a probe and silver staining showed that the NORs in this species are also located at one of the telomeric regions of both X chromosomes (Figure 3C,D). As in *A. fabae* a small DAPI negative region in one of the telomeres of the X chromosomes do not showed hybridization signals with this *mariner* (Figure 3E,F), and that this regions correspond with the NOR (Figure 3G,H). In summary, the results obtained clearly demonstrate that both *mariner* elements do not hybridize at the rDNA sites of their host species.

## 4. Discussion

Four full-length *mariner* transposable elements, belonging to the *Tc1-mariner* superfamily, have been found in several genera from different ant subfamilies [29,30,31]. Ants and aphids maintain a relationship of mutualism, with close physical contact, which could facilitate the HT of transposable elements between both groups of species. The aim of this study was to determine whether the four *mariner* elements found in the ant genomes were present in the genomes of two aphid species (*A. fabae* and *A. hederae*) and to analyse the possible existence of HT events.

According to our PCR assays, there are no *Sinvmar*, *Mboumar* and *Azteca mariner* elements in the genome of the two analysed *Aphis* species. However, sequence amplification has been obtained when PCR assays were performed using primers designed using the ITR sequences of the *Myrmar mariner* elements. We named these *mariners* as *Afabmar-Mr* and *Ahedmar-Mr*. These *mariner* elements show very low evolutionary divergence between them. In both species, full-length and internally deleted copies of the *mariners* were isolated. Half of the full-length copies of *A. fabae* have an ORF that could encode a transposase. The remaining sequences from this species and all *A. hederae* sequences do not have complete ORFs. The putative Afabmar-Mr active transposase conserved the catalytic D,D(34)D motif in the C-terminal domain. Similarly, the two highly conserved amino acid motifs, and other features of active *mariners* are also fully conserved in some copies and partially in others. All these characteristics suggest that *Afabmar-Mr* could be an active transposon. It cannot be ruled out that in *A. hederae* there are active copies and that by chance they have not been isolated. Unlike *Afabmar-Mr* sequences, the *mariner* sequences isolated from *T. ibericum*, *M. ruginodis* and *B. terrestris* were predictably inactive sequences [29,36,37]. 

The existence of *mariner*-like transposable elements have been reported in aphid genomes [55,56,57]. Recent studies on TEs have focused mainly on its general characterization and direct annotation of genomic data; most of the sequenced genomes are included in AphidBase (https://bipaa.genouest.org/is/aphidbase/, accessed on 22 October 2021). Unfortunately, the complete assemblies of the genome sequence of *A. fabae* and *A. hederae* are not available. Moreover, as we have commented previously, we have not found sequences with significant similarity in any other species of *Aphis* genus in any of the databases used.

However, the evolutionary pattern of *Ahedmar-Mr* and *Afabmar-Mr* shows some features shared with the *mariner* elements isolated from other different *Aphis* species [56]. In these species, *Mos1*-like elements were isolated in seven species belonging to the main tribes of the genus *Aphis*. All elements were non-active copies, with a high degree of sequence similarity, in clear discordance with the phylogenetic relationships of the host species. All of them are approximately 917 bp in length and are therefore defective copies. They share the same deletions, as it happens in all internally deleted copies isolated from *Aphis* species. This feature of the evolutionary pattern of *mariner* elements has been observed in other insects as well, since nucleotide deletions have been considered a common mechanism of the inactivation of TEs. For example, similar results have been reported in transposable elements isolated in ants. *Mboumar* is a *mariner* element isolated from several ant genomes [31]. In some species, such as *T. ibericum*, the authors find potentially active copies, whose putative transposases showed high sequence identity with the active Mboumar-9 transposase [48]. In the same species, inactive copies were also detected; all with the same 7-bp deletion that changes the reading frame and generates a premature stop codon. Furthermore, some copies isolated from other ant species shared this same deletion [31].

The use of molecular and morphological markers showed that *A. hederae* and *A. fabae* are phylogenetically closed species included in the “fabae group” [58,59]. All the obtained data in this study suggest that full-length and internally deleted *Myrmar*-like elements were present in the ancestor of both species, probably because of a previous HT event since this *mariner* is not present in other *Aphis* species. It is also possible that both species have suffered consecutive invasions by similar elements, although this hypothesis seems to be less plausible. After the HT event, the *mariner* would be transmitted by vertical transmission. According to the phylogenetic data, it does not seem likely that HT events have occurred directly from ants to aphids, at least between these *Aphis* species and the ant species in which *Myrmar mariner* elements have been studied. 

Nevertheless, the analysis of *Myrmar* elements in other species groups clearly suggests the involvement of HT events. The phylogenetic tree shows clustered in one clade the isolated *mariner* elements from the two *Aphis* species (Hemiptera), a beetle (Coleoptera), a butterfly (Lepidoptera) and a crustacean species. Although the sequences of each species were clustered together, the phylogeny of the *mariner* elements is in clear disagreement with that of its host species. In the second main clade, the isolated *mariner* sequences from eight different hymenopteran species form a supported subclade. Likewise, in almost all cases, the sequences of each species were clustered together in a well-supported clade and there is no concordance between the phylogeny of the *mariner* and its host hymenopteran species as we have detailed in the results section. *Myrmar* elements have been found in three species from the genus *Bombus*, but the sequences of each species are not clearly separated in the phylogenetic analysis, and the sequences from *B. campestris* and *B. sylvestris* are mixed (Supplementary Figure S4). Both are nearly species included in the subgenus *Psithyrus* [60]. Therefore, it is possible that both species show the *mariner* variability present in the common ancestor, without the necessary time having elapsed for species differentiation. The clustering found in *mariner* sequences from *D. vitifoliae* is also taxonomically discordant. *Daktulosphaira* and *Aphis* are the taxonomically closest genera whose *mariner* elements have been included in this paper. Both are Hemipteran and allocated to Sternorrhyncha suborder, although they belong to different families (Phylloxeridae and Aphididae, respectively). However, their *Myrmar* elements are not nearby in the phylogenetic tree. The availability and analysis of new assembled genomes of different aphid species will possibly provide new data on the evolution of this *mariner* element.

In summary, *Myrmar* elements have been isolated in species belonging to four insect orders (Hemiptera, Coleoptera, Lepidoptera and Hymenoptera) and in a crustacean species. In general, *mariner* sequences were clustered by species. This suggests that *mariner* elements are evolutionarily ancient, and that they have been transmitted vertically while diverging with the host species. However, the existence of HT events in the evolution of these *mariner* elements cannot be ruled out. Their presence in taxonomically and evolutionarily very distant species and the disagreement between the phylogeny of the *mariner* elements and the phylogeny of the hosts suggests the occurrence of this type of evolutionary events. Lorite et al. [29] have suggested the probable existence of an ancient HT process between the ant *M. ruginodis* and bumblebee *B. terrestris*. This idea is supported by the results obtained in the present study.

Horizontal transfer events between taxonomically distant species, as insects and crustaceans, have also been suggested by several authors [61,62]. For example, the horizontal spread of a *Tc1-mariner* element among Crustacean and several mosquito *Anopheles* species have been reported [62]. Equally, it has been suggested that up to 24% of an insect genome could have been generated by HT events, which indicates the importance of HT and TEs in the genome evolution of these organisms [63]. Recently, Gilbert et al. [28] have analysed the importance of TEs in the evolution of insects, taking into account the data available from the sequencing of numerous insect genomes. 

It has been reported that rDNA genes in aphids are located mainly in one telomere of each X chromosome [12,54,64]. A similar chromosome location of rDNA genes has been found in *A. fabae* by silver staining [53] and FISH using rDNA probes (this work). In *A. hederae*, rDNA genes have been localized for the first time using the two techniques. Therefore, in both species, the rDNA genes are located in the DAPI negative telomere region of each X chromosome. The hybridization signals in the homologous X chromosomes in both species showed small differences in their size, suggesting the existence of different numbers of rDNA genes in each telomere. This feature appears to be general in aphids rDNA genes clusters, due probably to a high incidence of mitotic recombination at this location [12]. In this study, we have also analysed the chromosomal location of *Ahedmar-Mr* and *Afabmar-Mr* elements. Both elements are present on all chromosomes, including X chromosomes. However, on the two X chromosomes of both species, no hybridization signals were detected on a small DAPI negative telomeric region, that corresponds with the rDNA sites.

The chromosomal location of *mariner* elements, widely distributed throughout the genome, as we have observed in these *Aphis* species, has also been found in numerous insect species and others organism. However, the absence of *Myrmar* elements in the rDNA genes is remarkable. For example, *Tnigmar-Mr*, also a *Myrmar*-like element, was widespread in the ant *T. ibericum* genome, including the rDNA sites [29]. However, in the same ant species, other DNA transposons are not located in the rDNA sites, but in the latter case, only some hybridization signals were observed on some chromosomes [29]. Nevertheless, *Tc1-mariner* coincident with rDNA chromosome sites has also been described. In these cases, it has been suggested that transposable elements may be involved in the evolution and dispersion in the genome of the rDNA genes [65]. In contrast, in the grasshopper *Eyprepocnemis plorans*, *mariner* elements and other types of transposable elements are absent in the pericentromeric region that contains rDNA genes [66]. Probably, as Amorim et al. [67] have recently pointed out, this variability and dispersion of data about the chromosomal location of TEs, especially in insects, is because studies focused on the differential accumulation of each type of TEs are still lacking. These analyses should be performed on the chromosomes of multiple individuals of the same species and/or related species [67]. 

To our knowledge, there are few studies about the chromosomal location of transposable elements in Aphidae, although some data about their characterization and annotation directly comes from genomic data [57]. In addition to the general considerations made on the chromosomal location of the elements of TEs, it is necessary to take into account the particularities of the aphids, and more specifically their X chromosomes, some already mentioned in the introduction. Several authors have reported the conservation of the (TTAGG)n telomeric sequence in all chromosomes of the genus *Aphis* [8,9,68]. However, the subtelomeric regions show a higher degree of polymorphism and a more variable composition, especially the X chromosomes [68]. Specifically, in several species of aphids, a subtelomeric satellite DNA of 169 bp has been found on all autosomal subtelomeric regions and at one end of the X chromosome but absent at the end that carries the NOR [12]. A non-LTR TRAS retrotransposon (identified as TRASAp1) has been detected in the aphids *Acyrthosiphon pisum* and *Myzus persicae* [10]. Using FISH techniques, the authors showed that TRAS elements were located near the telomeric repeats (TTAGG)n of all autosomes and on the X chromosome but only at the telomere opposite the one carrying the NOR. In addition to the above, the only CMA3 positive region (GC-rich heterochromatin) observed in the chromosomes of the aphids is limited to a single telomere of each X chromosome, coinciding with the NOR [10,12]. This region is clearly, and logically, DAPI negative (this work). In summary, the absence of repeated sequences, such as satellite DNA [68], retrotransposons [10] or *mariner* elements (this work) on the NORs of the X chromosomes has been observed. The structural differences between the two telomeres of the X chromosome could have some kind of relationship with the peculiar behaviour of the X chromosomes during sex determination. According to several authors, cited above, these differences could be related to the need for rDNA gene pairing, which is of great importance in determining sex in aphids, a process that is not yet fully understood.

As we have commented in the introduction, the aphid X chromosomes show high structural stability [19]. This long-term stability is surprising, according to the authors, due to the low level of gene expression, low level of selection of X-linked genes and accumulation of TEs. The authors suggest, among other hypotheses, that intact X chromosomes may be required for their proper removal during male determination. They also suggest that this conservation could be related to natural selection, or a process not yet known. Undoubtedly, new studies are needed to determine whether or not there are transposable elements in the rDNA sites and their relationship with the processes of sex determination in aphids, a process that still has great questions today.

## 5. Conclusions

*Myrmar mariner* elements isolated from *M. ruginodis* and *T. ibericum* ants have also been found in *A. fabae* and *A. hederae* (*Afabmar-Mr* and *Ahedmar-Mr* elements). Both elements are present on all chromosomes, including X chromosomes. However, on the two X chromosomes, no positive signals have been detected in one of the telomere regions of each X chromosome where the rDNA sites are found. The double FISH results demonstrate that these *mariner* elements do not hybridize to the rDNA sites of their host species. *Myrmar*-like elements are also present in other insect species as well as in one Crustacean species, suggesting the existence of ancient evolutionary events of horizontal transfer.

## Data Availability

Newly obtained sequences were deposited in GenBank, accession numbers OL441193 to OL441226.

## Supplementary Materials

The following are available online at https://www.mdpi.com/article/10.3390/genes12121966/s1, Table S1: Oligonucleotides used for *mariner* PCR amplifications, Figure S1: Agarose gel showing the PCR amplification of genomic DNA from *Aphis fabae* and *Aphis hederae* using primers based in the ITRs from *Myrmar* elements, Figure S2: Sequence alignment of all *Afabmar-Mr* and *Ahedmar-Mr rmariner* sequences, Figure S3: Sequence alignment of all putative Afabmar-Mr transposases, Figure S4: Maximum-likelihood analysis of the nucleotide *Myrmar-*like *mariner* sequences, Figure S5: Sequences found in the *Daktulosphaira vitifoliae* genome with similarity with the *Myrmar* elements.
