# Supplementary material for: Aphids and Ants, Mutualistic Species, Share a Mariner Element with an Unusual Location on Aphid Chromosomes
Source: Genes (Basel). 2021 Dec 9;12(12):1966. doi: 10.3390/genes12121966 (PMC8701394; doi:10.3390/genes12121966)
Supplement: Supplementary file 1 [file genes-12-01966-s001.zip › genes-1492721-supplementary.pdf]

SUPPLEMENTARY MATERIAL

Aphids and ants, mutualistic species, share a *mariner* element with an unusual location on aphid chromosomes

Vela, J., Montiel, E.E, Mora, P. Lorite, P., Palomeque, T.

Supplementary Table 1. Oligonucleotides used for *mariner* PCR amplifications.

mariner family	Oligonucleotide	Sequence (5' - 3')
Myrmar	Mrug-MAR	CCAGGTCTGTAAATATGAAACCGGAAT
Mboumar	ITR-MAR	CCAGGTGTGTCGGTAATTCCTTTCCGG
Sinvmar	Sinv-mar-1	TTAGGTGTTAAACTTAATTCCTGCCGCT
	Sinv-mar-2	AATTGAAGGTAACTTAATTCCTGCCGTT
Azteca	Azteca-1	CGAGGTTTGTTAAAAAAAAGGTGA
	Azteca-2	CGAGGTGTGATCAAAAAGTAAGGTGA
	Azteca-3	CGAGGTTTGTTAAAAAAAAAAAGTGA
	Azteca-4	CGAGGTGTGTTCAAAAAAAAGGTGA

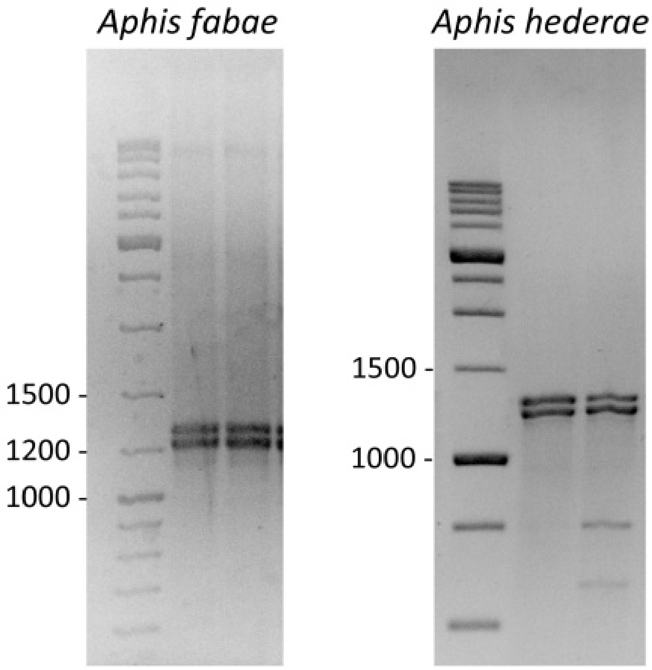

Figure S1. Agarose gel showing the PCR amplification of genomic DNA from *A. fabae* and *A. hederae* using primers based in the ITRs from *Myrmar* elements. The numbers indicate the size of DNA fragments in bp.

```
                       10        20        30        40        50        60        70        80        90       100
               ....|....|....|....|....|....|....|....|....|....|....|....|....|....|....|....|....|....|....|....|
AfabmarMr-50   CCAGGTCTGTAAATATGAAACCGGAATTTGCCCATAGGTGATTCTAGATGTCGAT CAGTTACCGACACATCACGTCAGCGGTGTCTCGTTATTGTATTG
AfabmarMr-54   .......................................................-............................................
AfabmarMr-57   .......................................................-............................................
AfabmarMr-13   .......................................................-.................T..........................
AfabmarMr-25   .......................................................-.................T..........................
AfabmarMr-51   .......................................................-.............G..............................
AfabmarMr-55   .......................................................-............................................
AfabmarMr-58   .......................................................-............................................
AfabmarMr-26   ............................C..........................-............................................
AfabmarMr-27   .......................................................-............................................
AfabmarMr-65   .......................................................G............................................
AfabmarMr-76   .......................................................G............................................
AfabmarMr-10   .......................................................G.................................C..........
AfabmarMr-96   .......................................................G............................................
AfabmarMr-59   .......................................................G............................................
AfabmarMr-94   ...............................................G.......G............................................
AfabmarMr-12   .......................................................G............................................
AfabmarMr-63   .......................................................G............................................
AfabmarMr-144  .......................................................G............................................
AhedmarMr-700  .......................................................-............................................
AhedmarMr-707  .......................................................-............................................
AhedmarMr-712  .......................................................-............................................
AhedmarMr-713  .......................................................-............................................
AhedmarMr-9    .......................................................G............................................
AhedmarMr-33   .......................................................G............................................
AhedmarMr-51   .......................................................G............................................
AhedmarMr-61   .......................................................G............................................
AhedmarMr-74   .......................................................G............................................
AhedmarMr-77   .......................................................G............................................
AhedmarMr-293  .......................................................G............................................
AhedmarMr-301  .......................................................G............................................
AhedmarMr-544  .......................................................G............................................
AhedmarMr-808  .......................................................G............................................
AhedmarMr-815  .................................G.....................G............................................
```

```
                      110       120       130       140       150       160       170       180       190       200
               ....|....|....|....|....|....|....|....|....|....|....|....|....|....|....|....|....|....|....|....|
AfabmarMr-50   ATATCCGACAAAGATTTCCAACTCACAATAACACGAGTTTGATTCAGTAGCATTAGTTTATTAGAGATAAACATGTCGAGTTTTGTGCCAACAAATTACG
AfabmarMr-54   ....................................................................................................
AfabmarMr-57   ....................................................................................................
AfabmarMr-13   ....................................................................................................
AfabmarMr-25   ....................................................................................................
AfabmarMr-51   ....................................................................................................
AfabmarMr-55   ....................................................................................................
AfabmarMr-58   ....................................................................................................
AfabmarMr-26   ....................................................................................................
AfabmarMr-27   ................................................................................C...................
AfabmarMr-65   ..........................................................C.........................................
AfabmarMr-76   ..........................................................C.........................................
AfabmarMr-10   ..........................................................C......................................G..
AfabmarMr-96   ..........................................................C.........................................
AfabmarMr-59   ..........................................................C.........................................
AfabmarMr-94   ..........................................................C.........................................
AfabmarMr-12   ..........................................................C.........................................
AfabmarMr-63   ..........................................................C.........................................
AfabmarMr-144  ..........................................................C.........................................
AhedmarMr-700  ....................................................................................................
AhedmarMr-707  ....................................................................................................
AhedmarMr-712  .......C............................................................................................
AhedmarMr-713  ..............................T.....................................................................
AhedmarMr-9    ..........................................................C.........................................
AhedmarMr-33   ..........................................................C.........................................
AhedmarMr-51   ..........................................................C.........................................
AhedmarMr-61   ..........................................................C.........................................
AhedmarMr-74   ..........................................................C.........................................
AhedmarMr-77   ..........................................................C.........................................
AhedmarMr-293  ..........................................................C.........................................
AhedmarMr-301  ..........................................................C.........................................
AhedmarMr-544  ..........................................................C.........................................
AhedmarMr-808  ..........................................................C.........................................
AhedmarMr-815  ..........................................................C.........................................
```

```
                       210       220       230       240       250       260       270       280       290       300
                  ....|....|....|....|....|....|....|....|....|....|....|....|....|....|....|....|....|....|....|....|
AfabmarMr-50      ATTTGCGGACAGCCCTACTTTTCTGTTTTCATCTGAAGAAAACCGCAGCGGAATCGCATCGAATGCTTCCGGAGGCATTTGGTGAGCATGCTCTCGGTAA
AfabmarMr-54      .....................................................................T..............................
AfabmarMr-57      .....................................................................T..............................
AfabmarMr-13      .....................................................................T..............................
AfabmarMr-25      ....................................................G................T..............................
AfabmarMr-51      .....................................................................T......G.......................
AfabmarMr-55      .....................................................................T..............................
AfabmarMr-58      .....................................................................T............C...............
AfabmarMr-26      ..............................G......................................T..............................
AfabmarMr-27      .....................................................................T..............................
AfabmarMr-65      ....T..............................G.................................T..............................
AfabmarMr-76      ....T..............................G.................................T........G.....................
AfabmarMr-10      ....T..............................G.................................T..............................
AfabmarMr-96      ....T..............................G.................................T..............................
AfabmarMr-59      ....T..............................G.................................T..............................
AfabmarMr-94      ....T..............................G.................................T..............................
AfabmarMr-12      ....T..............................G.................................T..............................
AfabmarMr-63      ....T..............................G...G.............................T..............................
AfabmarMr-144     ....T..............................G.................................T..............................
AhedmarMr-700     .....................................................................T............C...............
AhedmarMr-707     .............................................T.......................T............C...............
AhedmarMr-712     .....................................................................T............C...............
AhedmarMr-713     .....................................................................T............C...............
AhedmarMr-9       ....T.........................G....G.................................T..G...................C......
AhedmarMr-33      ....T.........................G....G.................................T..............................
AhedmarMr-51      ....T.........................G....G.................................T..............................
AhedmarMr-61      ....T.........................G....G.................................T..............................
AhedmarMr-74      ....T.........................G....G.................................T..............................
AhedmarMr-77      ....T.........................G....G.................................T..............................
AhedmarMr-293     ....T.........................G....G.................................T..............................
AhedmarMr-301     ....T.........................G....G.................................T..............................
AhedmarMr-544     ....T.........................G....G.................................T..............................
AhedmarMr-808     ....T.........................G....G.................................T..............................
AhedmarMr-815     ....T.........................G....G.................................T..............................

                       310       320       330       340       350       360       370       380       390       400
                  ....|....|....|....|....|....|....|....|....|....|....|....|....|....|....|....|....|....|....|....|
AfabmarMr-50      AACACAGTGTTTCGAGTGGTTTGAACGATTCAAAAGTGGCGATTTTGACGTGAGGAACGAAGAACGTGGTAGACCACCGAAAAAGTTTGAAGACGCTGAC
AfabmarMr-54      ....................................................................................................
AfabmarMr-57      ..........................................................................................G.........
AfabmarMr-13      .........................................................................T..........................
AfabmarMr-25      ....................................................................................................
AfabmarMr-51      ....................................................................................................
AfabmarMr-55      ....................................................................................................
AfabmarMr-58      ....................................................................................................
AfabmarMr-26      ...........................................C........................................................
AfabmarMr-27      ....................................................................................................
AfabmarMr-65      ......................A....G............................................G...........................
AfabmarMr-76      ......................A....G............................................G...........................
AfabmarMr-10      ......................A....G............................................G...........................
AfabmarMr-96      ......................A....G............................................G...........................
AfabmarMr-59      ......................A....G.........................T..................G...........................
AfabmarMr-94      ......................A....G............................................G...........................
AfabmarMr-12      ......................A....G............................................G...........................
AfabmarMr-63      ......................A....G............................................G...........................
AfabmarMr-144     ......................A....G............................................G...........................
AhedmarMr-700     .................................................................................G..................
AhedmarMr-707     ....................................................................................................
AhedmarMr-712     ....................................................................................................
AhedmarMr-713     ....................................................................................................
AhedmarMr-9       ......................A....G............................................G...........................
AhedmarMr-33      ......................A....G............................................G...........................
AhedmarMr-51      ......................A....G............................................G...........................
AhedmarMr-61      ......................A....G............................................G...........................
AhedmarMr-74      ......................A....G............................................G...........................
AhedmarMr-77      ......................A....G............................................G...........................
AhedmarMr-293     G.....................A....G............................................G...........................
AhedmarMr-301     ......................A....G............................................G............C..............
AhedmarMr-544     .............A........A....G............................................G...........................
AhedmarMr-808     ......................A....G............................................G...........................
AhedmarMr-815     ......................A....G............................................G...........................
```

```
                    410       420       430       440       450       460       470       480       490       500
                ....|....|....|....|....|....|....|....|....|....|....|....|....|....|....|....|....|....|....|....|
AfabmarMr-50    CTGCAAGCCTTGTTGGACGAAGACGACGCTCGAACACAACAAC---TGGCGGATCAGTTAAAGGTGACCCGGGAAGCCATCTCCCTTCGGTTGAAAGCCA
AfabmarMr-54    ...............................A......G....---......................................................
AfabmarMr-57    ...............................A...........---......................................................
AfabmarMr-13    ...............................A...........---......................................................
AfabmarMr-25    ...............................A...........---......................................................
AfabmarMr-51    ...............................A...........---......................................................
AfabmarMr-55    ...............................A...........---...................................C..................
AfabmarMr-58    ...............................A...........---......................................................
AfabmarMr-26    ...............................A...........---......................................................
AfabmarMr-27    ...............................A...........---......................................................
AfabmarMr-65    ...............................A...........AAC.............G...............................C........
AfabmarMr-76    ...............................AG..........AAC.............G........................................
AfabmarMr-10    ...............................A...........AAC.............G.....................C..................
AfabmarMr-96    ...............................A...........AAC......................................................
AfabmarMr-59    ...............................A...........AAC......................................................
AfabmarMr-94    ...............................A...........AAC......................................................
AfabmarMr-12    ...............................A...........AAC.................................................C....
AfabmarMr-63    ...............................A...........AAC......................................................
AfabmarMr-144   ...............................A...........AAC......................................................
AhedmarMr-700   ...............................A...........---......................................................
AhedmarMr-707   ...............................A...........---.....................................................T
AhedmarMr-712   ...............................A...........---......................................................
AhedmarMr-713   ...............................A...........---......................................................
AhedmarMr-9     ...............................A...........AAC......................................................
AhedmarMr-33    ...............................A...........AAC......................................................
AhedmarMr-51    ...............................A...........AAC......................................................
AhedmarMr-61    ...............................A...........AAC......................................................
AhedmarMr-74    ...............................A...........AAC......................................................
AhedmarMr-77    ...............................A...........AAC......................................................
AhedmarMr-293   ...............................A...........AAC..................G...................................
AhedmarMr-301   ...............................A...........AAC......................................................
AhedmarMr-544   ...............................A...........AAC......................................................
AhedmarMr-808   ...............................A...........AAC......................................................
AhedmarMr-815   ...............................A...........AAC......................................................

                    510       520       530       540       550       560       570       580       590       600
                ....|....|....|....|....|....|....|....|....|....|....|....|....|....|....|....|....|....|....|....|
AfabmarMr-50    TGGGAAAGATACAAAAGGCGGGGAAATGGGTTCCACATGAACTCAACGAAAGACAGCAGGAAAACCGCAAAACATCATGCGAAATGCTGCTCGCCAGGTT
AfabmarMr-54    ....................................................................................................
AfabmarMr-57    ....................................................................................................
AfabmarMr-13    .....................................................................G..............................
AfabmarMr-25    ....................................................................................................
AfabmarMr-51    ....................................................................................................
AfabmarMr-55    ....................................................................................................
AfabmarMr-58    .........................-..........................................................................
AfabmarMr-26    ....................................................................................................
AfabmarMr-27    ..................................................................................................C.
AfabmarMr-65    ....................................................................................................
AfabmarMr-76    ....................................................................................................
AfabmarMr-10    ....................................................................................................
AfabmarMr-96    ...................................................................................................C
AfabmarMr-59    ....................................................................................................
AfabmarMr-94    ....................................................................................................
AfabmarMr-12    ....................................................................................................
AfabmarMr-63    ....................................................................................................
AfabmarMr-144   ....................................................................................................
AhedmarMr-700   .........................-....................................G...........C.........................
AhedmarMr-707   .........................-..........................................................................
AhedmarMr-712   .........................-..........................................................................
AhedmarMr-713   .........................-..........................................................................
AhedmarMr-9     ....................................................................................................
AhedmarMr-33    ....................................................................................................
AhedmarMr-51    ....................................................................................................
AhedmarMr-61    .....................................................................T..............................
AhedmarMr-74    ....................................................................................................
AhedmarMr-77    .............................................................G......................................
AhedmarMr-293   ....................................................................................................
AhedmarMr-301   ....................................................................................................
AhedmarMr-544   ....................................................................................................
AhedmarMr-808   ....................................................................................................
AhedmarMr-815   .........................................T...................G......................................
```

```
                      610       620       630       640       650       660       670       680       690       700
              ....|....|....|....|....|....|....|....|....|....|....|....|....|....|....|....|....|....|....|....|
AfabmarMr-50  CAAAAGAAAGTCATTTCTCCATCGAATTGTGACTGGCGATGAAAAGTGGATATATTTCGAGAATCCTAAGCGCAAAAAGTCATGGGTTACTCCGGGTGAA
AfabmarMr-54  ....................................................................................................
AfabmarMr-57  ....................................................................................................
AfabmarMr-13  ....................................................................................................
AfabmarMr-25  ....................................................................................................
AfabmarMr-51  ....................................................................................................
AfabmarMr-55  ....................................................................................................
AfabmarMr-58  ..............C.....................................................................................
AfabmarMr-26  ....................................................................................................
AfabmarMr-27  .............................................................................G......................
AfabmarMr-65  ....................................................................................................
AfabmarMr-76  ................................................................................................G...
AfabmarMr-10  ....................................................................................................
AfabmarMr-96  ....................................................................................................
AfabmarMr-59  ....................................................................................................
AfabmarMr-94  ....................................................................................................
AfabmarMr-12  ....................................................................................................
AfabmarMr-63  ....................................................................................................
AfabmarMr-144 ............................A.......................................................................
AhedmarMr-700 ..............C.....................................................................................
AhedmarMr-707 ..............C.......................................C.............................................
AhedmarMr-712 ..............C.....................................................................................
AhedmarMr-713 ..............C.....................................................................................
AhedmarMr-9   ....................................................................................................
AhedmarMr-33  ....................................................................................................
AhedmarMr-51  ..................................A.................................................................
AhedmarMr-61  ....................................................................................................
AhedmarMr-74  ....................................................................................................
AhedmarMr-77  ....................................................................................................
AhedmarMr-293 ....................................................................................................
AhedmarMr-301 ...........................................................................G........................
AhedmarMr-544 ....................................................................................................
AhedmarMr-808 ....................................................................................................
AhedmarMr-815 ....................................................................................................

                      710       720       730       740       750       760       770       780       790       800
              ....|....|....|....|....|....|....|....|....|....|....|....|....|....|....|....|....|....|....|....|
AfabmarMr-50  CCATCAACATCGACGGCAAGACCAAACCGTTACGGACGAAAGACAATGCTGTGTGTTTGGTGGGATCAGAAGGGCGTAATCTACTACGAGCTGCTGAAAC
AfabmarMr-54  ....................................................................................................
AfabmarMr-57  ....................................................................................................
AfabmarMr-13  ....................................................................................................
AfabmarMr-25  ......G.............................................................................................
AfabmarMr-51  ....................................................................................................
AfabmarMr-55  ....................................................................................................
AfabmarMr-58  ....................................................................................................
AfabmarMr-26  ....................................................................................................
AfabmarMr-27  ....................................................................................................
AfabmarMr-65  .............................C..T.....G.............................................................
AfabmarMr-76  .............................C..T.....G.............................................................
AfabmarMr-10  .............................C..T.....G.............................................................
AfabmarMr-96  .............................C..T.....G.............................................................
AfabmarMr-59  .............................C..T.....G.............................................................
AfabmarMr-94  .............................C..T.....G.............................................................
AfabmarMr-12  .............................C..T.....G.............................................................
AfabmarMr-63  .............................C..T.....G.............................................................
AfabmarMr-144 .............................C..T.....G.............................................................
AhedmarMr-700 ....................................................................................................
AhedmarMr-707 ...................................G................................................................
AhedmarMr-712 ....................................................................................................
AhedmarMr-713 ....................................................................................................
AhedmarMr-9   .............................C..T.....G.............................................................
AhedmarMr-33  .............................C..T.....G............C...............................................
AhedmarMr-51  .............................C..T.....G.............................................................
AhedmarMr-61  .............................C..T.....G.............................................................
AhedmarMr-74  .............................C..T.....G.............................................................
AhedmarMr-77  .............................C..T.....G.............................................................
AhedmarMr-293 .............................C..T.....G............................G................................
AhedmarMr-301 .............................C..T.....G.............................................................
AhedmarMr-544 .............................C..T.....G............C...............................................
AhedmarMr-808 .............................C..T.....G.............................................................
AhedmarMr-815 .............................C..T.....G..................................A..........................
```

```
                       810       820       830       840       850       860       870       880       890       900
              ....|....|....|....|....|....|....|....|....|....|....|....|....|....|....|....|....|....|....|....|
AfabmarMr-50  CTGGGGAAACTGTGAATACTCAACGCTATCGACAACAAATGACCGATTTGGATCGAGTTTTGCGTGAAAAACGACCAGAATACCAACAAAGACAACACAA
AfabmarMr-54  ....................................................................................................
AfabmarMr-57  ....................................................................................................
AfabmarMr-13  ....................................................................................................
AfabmarMr-25  ....................................................................G...............................
AfabmarMr-51  ....................................................................................................
AfabmarMr-55  ....................................................................................................
AfabmarMr-58  ....................................................................................................
AfabmarMr-26  ....................................................................................................
AfabmarMr-27  ....................................................................................................
AfabmarMr-65  ..................................................................G.................................
AfabmarMr-76  ..................................................................G.................................
AfabmarMr-10  ..................................................................G.................................
AfabmarMr-96  ....................................................................................................
AfabmarMr-59  ....................................................................................................
AfabmarMr-94  ....................................................................................................
AfabmarMr-12  ....................................................................................................
AfabmarMr-63  ....................................................................................................
AfabmarMr-144 ....................................................................................................
AhedmarMr-700 ....................................................................................................
AhedmarMr-707 ....................................................................................................
AhedmarMr-712 .....................................................................G..............................
AhedmarMr-713 .....................................................................G..............................
AhedmarMr-9   ....................................................................................................
AhedmarMr-33  ....................................................................................................
AhedmarMr-51  ...................................................................G................................
AhedmarMr-61  ....................................................................................................
AhedmarMr-74  ....................................................................................................
AhedmarMr-77  ....................................................................................................
AhedmarMr-293 ....................................................................................................
AhedmarMr-301 ....................................................................................................
AhedmarMr-544 ....................................................................................................
AhedmarMr-808 ....................................................................................................
AhedmarMr-815 ....................................................................................................

                       910       920       930       940       950       960       970       980       990      1000
              ....|....|....|....|....|....|....|....|....|....|....|....|....|....|....|....|....|....|....|....|
AfabmarMr-50  GGTGATTTTGCTGCATGACAATGCACCAGCACACAAAGCAAAACTGGTCCAGGAAACGATAGAGTCGTTCCGTTGGAAGATTCTTCAGCACGCGGCTTAC
AfabmarMr-54  ....................................................................................................
AfabmarMr-57  ....................................................................................................
AfabmarMr-13  ............................................................................G.......................
AfabmarMr-25  ............................................................................G.......................
AfabmarMr-51  ...............................T............................................G...............TT......
AfabmarMr-55  ...............................T............................................G...............TT......
AfabmarMr-58  ...............................T............................................G...............TT......
AfabmarMr-26  ...............................T............................................G...............TT......
AfabmarMr-27  ...............................T............................................G...............TT......
AfabmarMr-65  ............................................................................G.......................
AfabmarMr-76  ............................................................................G.......................
AfabmarMr-10  ............................................................................G.......................
AfabmarMr-96  ............................................................................G.......................
AfabmarMr-59  ............................................................................G.......................
AfabmarMr-94  ............................................................................G.......................
AfabmarMr-12  ............................................................................G.......................
AfabmarMr-63  ...............................................................A............G.......................
AfabmarMr-144 ...........C................................................................G.......................
AhedmarMr-700 ...............................T............................................G...............TT......
AhedmarMr-707 ...............................T............................................G...............TT..C...
AhedmarMr-712 ...............................T............................................G...............TT......
AhedmarMr-713 ...............................T............................................G...............TT......
AhedmarMr-9   ............................................................................G.......................
AhedmarMr-33  ............................................................................G.......................
AhedmarMr-51  ............................................................................G.......................
AhedmarMr-61  ............................................................................G.......................
AhedmarMr-74  ............................................................................G.......................
AhedmarMr-77  ............................................................................G.......................
AhedmarMr-293 ............................................................................G.......................
AhedmarMr-301 ............................................................................G.......................
AhedmarMr-544 ............................................................................G.......................
AhedmarMr-808 ............................................................................G.......................
AhedmarMr-815 ............................................................................G.......................
```

```
                1010      1020      1030      1040      1050      1060      1070      1080      1090      1100
            ....|....|....|....|....|....|....|....|....|....|....|....|....|....|....|....|....|....|....|....|
AfabmarMr-50  TCACCGGACCTTGCCCCATCCGATTACTACTTGTTTGCATCGATGGGACACGCACTTGCTGAGCAGCGCTTTACTTCTTACGAAGATGTACGAAAATGGC
AfabmarMr-54  ....................................................................................................
AfabmarMr-57  ....................................................................................................
AfabmarMr-13  ....................................................................................................
AfabmarMr-25  ....................................................................................................
AfabmarMr-51  ....................................................................................................
AfabmarMr-55  ....................................................................................................
AfabmarMr-58  ....................................................................................................
AfabmarMr-26  .....................................T..............................................................
AfabmarMr-27  ....................................................................................................
AfabmarMr-65  .....................................................................................G..............
AfabmarMr-76  .....................................................................................G..............
AfabmarMr-10  .....................................................................................G..............
AfabmarMr-96  .....................................................................................G..............
AfabmarMr-59  .....................................................................................G..............
AfabmarMr-94  .....................................................................................G..............
AfabmarMr-12  .....................................................................................G..............
AfabmarMr-63  ...............................C.....................................................G..............
AfabmarMr-144 .....................................................................................G..............
AhedmarMr-700 ....................................................................................................
AhedmarMr-707 ....................................................................................................
AhedmarMr-712 ....................................................................................................
AhedmarMr-713 ....................................................................................................
AhedmarMr-9   .....................................................................................G..............
AhedmarMr-33  .....................................................................................G..............
AhedmarMr-51  .....................................................................................G..............
AhedmarMr-61  .....................................................................................G..............
AhedmarMr-74  .....................................................................................G..............
AhedmarMr-77  .....................................................................................G..............
AhedmarMr-293 .....................................................................................G..........C...
AhedmarMr-301 .....................................................................................G..............
AhedmarMr-544 .....................................................................................G..............
AhedmarMr-808 .....................................................................................G..............
AhedmarMr-815 .....................................................................................G..............

                1110      1120      1130      1140      1150      1160      1170      1180      1190      1200
            ....|....|....|....|....|....|....|....|....|....|....|....|....|....|....|....|....|....|....|....|
AfabmarMr-50  TCGATGACTGGTTTGCCTCAAAAGAGCAACAGTTCTTTTGGCGCGGAATCCACAAATTGCCAGGAAGATGGGAAAAATGTATAGATAGCGACGGACAATA
AfabmarMr-54  ...................G...........................................A....................................
AfabmarMr-57  ...............................................................A....................................
AfabmarMr-13  ...................................................G...........A....................................
AfabmarMr-25  ...............................................................A....................................
AfabmarMr-51  .............C.............................T...................A....................................
AfabmarMr-55  ...........................................T...................A....................................
AfabmarMr-58  ...........................................T...................A....................................
AfabmarMr-26  ...........................................T...................A....................................
AfabmarMr-27  ...........................................T...................A....................................
AfabmarMr-65  .........................................A......----------------------------------------------------
AfabmarMr-76  .........................................A......----------------------------------------------------
AfabmarMr-10  .........................................A......----------------------------------------------------
AfabmarMr-96  .........................................A......----------------------------------------------------
AfabmarMr-59  .........................................A......----------------------------------------------------
AfabmarMr-94  .........................................A......----------------------------------------------------
AfabmarMr-12  .........................................A......----------------------------------------------------
AfabmarMr-63  .........................................A......----------------------------------------------------
AfabmarMr-144 .........................................A......----------------------------------------------------
AhedmarMr-700 .......................................A...T...................A....................................
AhedmarMr-707 ...........................................T...................A....................................
AhedmarMr-712 ...........................................T...................A....................................
AhedmarMr-713 ...........................................T...................A....................................
AhedmarMr-9   .........................................A......----------------------------------------------------
AhedmarMr-33  .........................................A......----------------------------------------------------
AhedmarMr-51  .........................................A......----------------------------------------------------
AhedmarMr-61  .........................................A......----------------------------------------------------
AhedmarMr-74  .........................................A......----------------------------------------------------
AhedmarMr-77  .........................................A......----------------------------------------------------
AhedmarMr-293 .........................................A......----------------------------------------------------
AhedmarMr-301 .........................................A......----------------------------------------------------
AhedmarMr-544 .........................................A......----------------------------------------------------
AhedmarMr-808 .........................................A......----------------------------------------------------
AhedmarMr-815 .........................................A......----------------------------------------------------
```

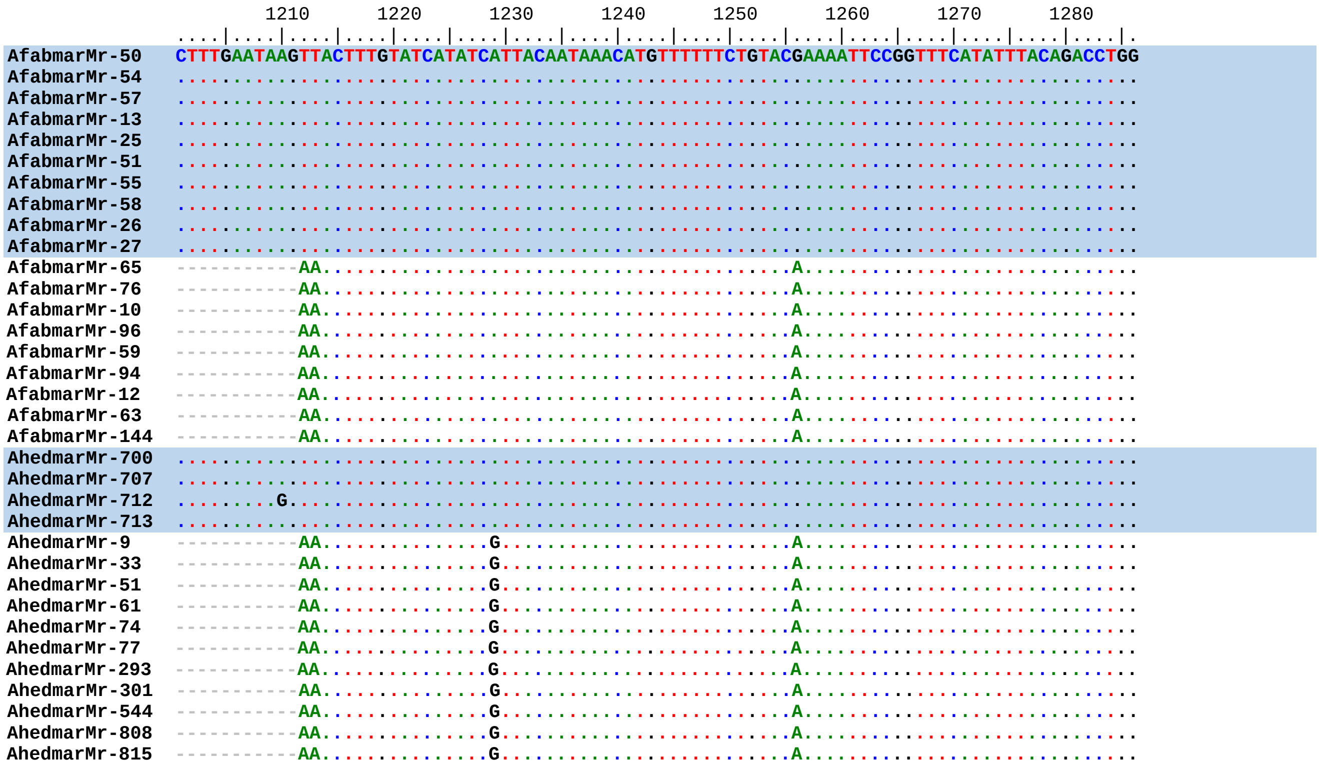

Figure S2. Sequence alignment of all *Afabmar-Mr* and *Ahedmar-Mr rmariner* sequences. Full-length copies of *mariners* in both species are marked with blue shading.

```
                     10        20        30        40        50        60        70        80        90       100
              ....|....|....|....|....|....|....|....|....|....|....|....|....|....|....|....|....|....|....|....|
                                                                                                   HTH motif
AfabmarMr-13  MSSFVPTNYDLRTALLFCFHLKKTAAESHRMLLEAFGEHALGKTQCFEWFERFKSGDFDVRNEERGRSPKKFEDADLQALLDEDDAQTQQLADQLKVTRE
AfabmarMr-25  ...................................................................P................................
AfabmarMr-26  ...................R.....................................L.........P................................
AfabmarMr-27  ...................................................................P................................
AfabmarMr-50  ................................P..................................P..................R.............
AfabmarMr-51  ...................................................................P................................
AfabmarMr-54  ...................................................................P................................
AfabmarMr-55  ...................................................................P................................
AfabmarMr-57  ...................................................................P................................

                    110       120       130       140       150       160       170       180       190       200
              ....|....|....|....|....|....|....|....|....|....|....|....|....|....|....|....|....|....|....|....|
                                 WVPHEL                            D
AfabmarMr-13  AISLRLKAMGKIQKAGKWVPHELNERQQENRRTSCEMLLARFKRKSFLHRIVTGDEKWIYFENPKRKKSWVTPGEPSTSTARPNRYGRKTMLCVWWDQKG
AfabmarMr-25  ...............................K.............................................A......................
AfabmarMr-26  ....................................................................................................
AfabmarMr-27  ...............................K.........L.........................R................................
AfabmarMr-50  ...............................K....................................................................
AfabmarMr-51  ...............................K....................................................................
AfabmarMr-54  ...............................K....................................................................
AfabmarMr-55  ..P............................K....................................................................
AfabmarMr-57  ...............................K....................................................................

                    210       220       230       240       250       260       270       280       290       300
              ....|....|....|....|....|....|....|....|....|....|....|....|....|....|....|....|....|....|....|....|
                                                             D                                  YSPDLAP  D
AfabmarMr-13  VIYYELLKPGETVNTQRYRQQMTDLDRVLREKRPEYQQRQHKVILLHDNAPAHKAKLVQETIESFRWEILQHAAYSPDLAPSDYYLFASMGHALAEQRFT
AfabmarMr-25  ...............................E....................................................................
AfabmarMr-26  ....................................................Y...................V..............V............
AfabmarMr-27  ....................................................Y...................V...........................
AfabmarMr-50  ...................................................................K................................
AfabmarMr-51  ....................................................Y...................V...........................
AfabmarMr-54  ...................................................................K................................
AfabmarMr-55  ....................................................Y...................V...........................
AfabmarMr-57  ...................................................................K................................

                    310       320       330       340
              ....|....|....|....|....|....|....|....
AfabmarMr-13  SYEDVRKWLDDWFASKEQQFFWRGIRKLPERWEKCIDSDGQYFE
AfabmarMr-25  .........................H..................
AfabmarMr-26  .........................H..................
AfabmarMr-27  .........................H..................
AfabmarMr-50  .........................H...G..............
AfabmarMr-51  .........................H..................
AfabmarMr-54  .........................H..................
AfabmarMr-55  .........................H..................
AfabmarMr-57  .........................H..................
```

Figure S3. Sequence alignment of all putative *Afabmar-Mr* transposases. The conserved D,D(34)D motif, the also conserved *mariner* transposase WVPHEL and YSPDL motifs and the HTH motif are shown in different colors (yellow, green and blue respectively).

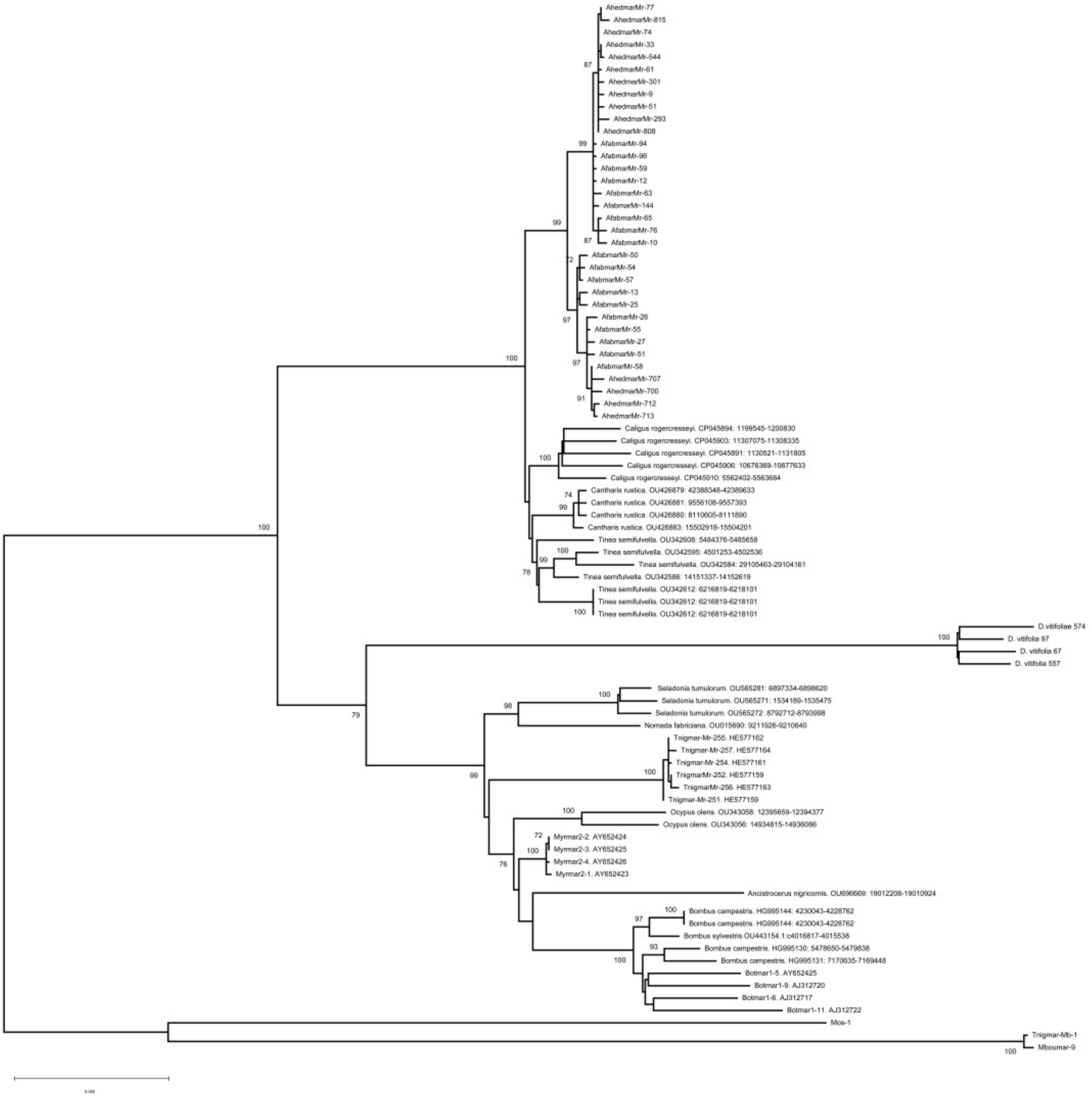

Figure S4. Maximum-likelihood analysis of the nucleotide *Myrmar* like *mariner* sequences. The accession numbers and nucleotide position of each *mariner* element are indicated. The data corresponding to the sequences of *Daktulosphaira vitifoliae* are shown in Figure S5. Numbers indicate the bootstrap values over 1000 replications. Only bootstrap support values greater than 70% are showed.

```
>scaffold67 Apollo scaffold67:195087..196370 (+ strand) length=1284
CCAGGTGTGTAAATATGAAACCGGAATTTGCCGATAGGTGACACTACCTATTGATACCGTAACCCATTTTCACTTGTATTGTGTCGTGTTGTTTGGGTGAC
TTATGCGCACGAGTTCCAACTCGTACCAGCGAAAGTTTCATTTAGTAGTGTAGTGAGGCTCGTGTGAAACATGTCGAAGTTTGTGCCAAGAAAGTATGATT
TGCGGACAGCGTTGATCTTTTGTTTCCATCTGAAGAAAACTGCATCAGAATCGCATCAAATGCTTGTGGAAGCTTACGGTGAGCATGCTCTGGGAAAGTCA
CAGTGTTTTGAGTGGTTCAAGAAATTTAAAAGTGGCGATTTCGATGTGAGGGATGCAGAACGTGGTCGACCACCGAAAAAGTTTGAAGATAATGAGTTGCA
AGCGTTGTTGGATGAGGATGCAACACAAACCCAAAAACAACTTGCAGATCAATTGAATGTGACCCGGGACGCCGTATCCATTCGTTTGAAAGCCATGGGAA
AGATCCAAAAGGTTGGAAAATGGGTTCCACATCAACTGACGGAAAGGCAACGGGAAAACTGACAAACCATTTGCGAAATGCTGATCGCCAGGCACAGAATA
AAATCTTTTCTCCATTACATTGTAACTGGCGATGAAAAATGGATATATTTTGAGAATCCTAAGCGACAAAAATCATGGGTAGCTCCAGGAGAACAATCAAA
GTCGAGTGCGAGGCCAAATCGCTTTGGAAAGAAAACAATGCTCTGTGTTTGGTGGGATCAGAAGGGTGTTATCTATTATGAGCTTCTTAAACCTGGCGAAA
CTGTCAACACTGAACGTTACCTACAACAACTATCAAATTTGTTTCAAGCTTTGGAAGACAAACGGCCGGAACTTCAACGACGGCAACACCAGGTGATTTTG
CTTTACGACAATACTCCGGCACACACGGCTAAAGTGGTCAAGGAATTAATCGAGGCGTTTGGTTGGGAGACTTTATCGCATGCCGCTTATTCGCCAGACTT
GGCTTCTTCAGACTACCATTTATTTGCATCTTTGGGACACGCACTCGCTGAGCAGCGCTTCTCTTCCTACGAAGATGTGCAAAAATAGTTCGACAATTGGT
TTGCGTCAAAAGAGCAACAATTCTTCTGGCGTGGAATCCATAAATTGCCCGAAAGATGGGAAAAATGTATAAGTAGCGAAGGGCAATACTTTGAATAGGAT
GTATAGTATCA GTCTTGTACAATAAACTAGTATTTTATGTCAAATAATTCCGGTTTCATATTTACACACCTGG

>scaffold557 Apollo scaffold557:100292..101408 (+ strand) length=1117
AAACATGTCGAAGTTTGTGCCAGGAAAGTATGATTTGCGGACAGCGTTGATCTTCTGTTTCCATCCGAAGAAAACTGCGTCAAAATCGCATCAAATGCTTG
TGGAAGCTTACGGTGAGCATGCTCTGGGGAAGTCACAGTGTTTTGAGTGGTTCAAGAAATTTAAAAGTGGCGATTTCGATGTGAGGGATGCAGAACGAAGT
CGACCACCGAAAAAGTTTGAAGACAACGAGTTGCAAGCGTTGTTGGATGAGGATGCAACGCAAACCCAAAAACAACTTGCAGATCAATTGAATGTGACCCG
GGAAGCCGTATCCATTCGTTTGAAAGCCATGGGAAAGATCCAAAAGGTGGGAAAATGGGTTCCACATCAACTGACGGAAAGGCAACGGGAAAACCGACAAA
CCACTTGTGAAATGCTGCTCGCCAGGCACAGAATAAAATCTTTTCTCCATCGCATTGTAACTGGCGATGAAAAATGGATATATTTTGATAATCCTAAGCGA
CAAAAATCATGGGTAGCTCCAGGAGAACCATCAAAGTCGAGTGCGAGGCCAAATCGCTTTGGAAAGAAAACAATGCTCTATGTTTGGTGGGATTAGAAGGG
TGTTATCTATTATGAGCTTCTTAAACCTGGCGAAACTGTCAATACTGAACGTTACCTACAACAACTGTCAAATGTGTTTCAAGCTTTGGAAGACAAACGGC
CGGAACTTCAACGACGGCAACACCAGGTGATTTTGCTTCACGACAATGCTCCGGCACACAGGGCTAAAGTGGTTTAGGAATTAATCGAGGCGTTTGGTTGG
GAGACTTTATCGCATGCCGCTTATTCGCCAGACTTGGCTCCTTCAGACTACCATTTATTTGCATCTTTGGGACACGTACTCGCTGAGCAGCGCTTTTTTTC
CTACGAAGATGTGCAAAAATAGCTCGACAATTGGTTTGCGTCAAAAGAGCAACAATTCTTCTGGCGTGGAATCCATAAATTGCCCGAAAGATGGGAAAAAT
GTATAAGTAGCGAAGGGCAATACTTCGAATAGGATGTATAGTATCAGTTTTGTACGATAAACTAGTATTTTATGTCAAATAATTCCGGTTTCATATTTACA
CACCTGG

>scaffold97 Apollo scaffold97:110013..111289 (- strand) class=match length=1277
CCAGGTGTGTAAATATGAAACCGGAATTTGCCGATAGGTGACGCTACCTATTGATACCGTAACCCATTTTCACTTGTATTGTGTCGTGTTGTTTGGGTGAC
TTATGCGCACGAGTTCCAACTCGTACCAGCGAAAGTTTCATTTAGTAGTGTAGTGAGGCTCGTGTGAAACATGTCGAAGTTTGTGCCAGGAAAGTATGATT
TGCGGACAGCGTTGATCTTCTGTTTCCATCTGAAGAAAACTGCGTCAGAATCGCATCAAATGCTTGTGGAAGCTTACGGTGAGCATGCTCTGGGGAAGTCA
CAGTGTTTTGAGTGGTTCAAGAAATTTAAAAGTGGCGATTTCGATGTGAGGGATGCAGAACGTGGTCGACCACCGAAAAAGTTTGAAGACAACGAGTTGCA
AGCGTTGTTGGATGAGGATGCAACGCAAACCCAAAAACAACTTGCAGATCAATTGAATGTGACCCGGGAAGCCGTATCCATTCGTTTGAAAGCCATGGGAA
AGGTGGGAAAATGGGTTCCACATCAACTGACGGAAAGGCAACGGGAAAACCGACAAACCACTTGCGAAATGCTGCTCGCCGGGCACAGAATAAAATCTTTT
CTCCATCGCATTGTAACTGGCGATGAAAAATGGATATATTTTGAGAATCCTAAGAAACAAAAATCATGGGTAGAGCTCCAGGAGAACCATCAAAGTCGAGT
GCGAGGCCAAATCGCTTTGGAAAGAAAACAATGCTCTGTGTTTGGTGAAATCAGAAGGGTGTTATCTATTATGAGCTTCTTAAACCTGGCGAAACTGTCAA
CACTGAACGTTACCTACAACAATTGTCAAATTTGTTTCAAGCTTTGGAAGACAAACGACCGGAACTTCAACGACGACAACACCAGGTGATTTTGCTTCATG
ACAATGCTCTGGCACACATGGCTAAAGTGGTCAAGGAATTAATCGATGTGTTTGGTTGGGAGACTTTATCGTATGCCGCTTATTCGCCAGACTTGGCTCCT
TCAGAATACCATTTATTTGCATCTTTGGGACACGCACTCGCTGCGCAGCGCTTCTCTTCCTACAGAGATGTGCAAAAATGGCTCGACAATTGGTTTGCGTC
AAAAGAGCAACAATTCTTCTGGCGTGGAATCCATAAATTGCCCGAAAGATGGGAAAAATGTATAAGTAGCGAAGGGCAATACTTCGAATAGGATGTATAGT
ATCAGTTTTGTACAATAAACTAGTATTTTATGTCAAATAATTCCGGTTTCATATTTACACACCTGG

>scaffold574 Apollo scaffold574:127835..128944 (+ strand) class=match length=1270
AAACATGTCGAAGTTTGTGCCAGGAAAGTATGATTTGCGGACAGCGTTGATCTTCTGTTTCCATTTAAAGAAAACTGCGTCAGAATCGCATCAAATGCTTG
TGGAAGCTTACGGTGAGCATGCTCTGGGAGAGTCACAGTATTTTGAGTGGTTGAAGAAATTTAAAAGTGGCGATTTCGATGTGAGGGATACAAAACGTGGT
CGACCACCGAAAAAGTTTGAAGACAACGAGTTGCAAGCGTTGTTGGATGAGGATGCAACGCAAACCCAAAAACAACTTGCAGATCAATTGAATGTGACCCG
GGAAGCCGTATCCATTCGTTTGAAAGCCATGGGAAAGATCCAAAAGGTGGAAAAATGGGTTCCACATCAACTGACGGAAAGGCAATGGGAAAACCGACAAA
CCACTTGCGAAATGCTGCTCGCCAGGCACAGAATAAAATCTTTTCTCCATCGCATTGTATCTGGCGATGAAAAATGGATATATTTTGAGAATCCTAAGCAA
CAAAAATCATGGGTAGCTCCAGGAGAACCTTCAAAGTCGAGTGCGAGGCCAAATCGCTTTGGAAAGAAAACAATGCTCTGTGTTTGATGGGATCAGAAGGG
TGTTATCTATTATGAGCTTCTTAAACCTGGCGAAACTGTCAACACTGAACGTTACCTACAACAACTGTCAAATTTGTTTCAAGCTTTGGAAGACAAACGGC
CCGAACTTCAACGACGGGAACAGCAGATGATTTTGCTTCACGACAATGCTCCGGCACACACGGCTAAAGTGGTCAAATCGAGGCGTTAGGTTGGGAGACTT
TATGGCATGCCGCTTATTCGCCAGACTTGGCTCCTTCAGACTACCATTTATTTGCATCTTTGGGACACGCAATCGCTGAGCAGCGCTTCTCTTCCTACGAA
GATGTGCAAAAATGGCTCGAAAATTGGTTTGCGTCAAAAGAGCAAGAATTCTTCTGGCGTGGAATCCATAAATTGCCCTAAAGATGGGAAAAATGTATAAG
TAGCGAAGGGCAATACTTCGAATGGGATGTATAGTATCAGTTTTGTACAATAAACTAGTATTTTATGTCAAATAATTCCGGTTTCATATTTACATACCTGG
```

Figure S5. Sequences found in the *Daktulosphaira vitifoliae* genome with similarity with the *Myrmar* elements. These sequences were obtained from the AphidBase web server (https://bipaa.genouest.org/is/aphidbase/).
